# Lethal variants in humans: lessons learned from a large molecular autopsy cohort

**DOI:** 10.1186/s13073-021-00973-0

**Published:** 2021-10-13

**Authors:** Hanan E. Shamseldin, Lama AlAbdi, Sateesh Maddirevula, Hessa S. Alsaif, Fatema Alzahrani, Nour Ewida, Mais Hashem, Firdous Abdulwahab, Omar Abuyousef, Hiroyuki Kuwahara, Xin Gao, Molecular Autopsy Consortium, Hesham Aldhalaan, Abdullah Alfaifi, Amal Alhashem, Khalid Alhasan, Maha Alnemer, Nada Alsahan, Suad Alyamani, Hamad Alzaidan, Kerr Brownyn, Angela Condie, Eissa Faqeih, Rubina Khan, Wesam Kurdi, Osama Muthaffar, Amira Nabil, William Newman, Mohammad M. Al-Qattan, Zuhair Rahbeeni, Rawda Sunbul, Mohamad-Hani Temsah, Maha Tulbah, Mohammed Zain, Fowzan S. Alkuraya

**Affiliations:** 1 Department of Translational Genomics, Center for Genomic Medicine, King Faisal Specialist Hospital and Research Center, Riyadh, Saudi Arabia; 2 Department of Zoology, College of Science, King Saud University, Riyadh, Saudi Arabia; 3 Center of Excellence for Biomedicine, King Abdulaziz City for Science and Technology, Riyadh, 12354 Saudi Arabia; 4 Computational Bioscience Research Center (CBRC), King Abdullah University of Science and Technology (KAUST), Thuwal, Saudi Arabia

**Keywords:** EHBP1L1, BMPR1A, MSN, FAAH2, Embryonic lethal, founder, loss of function, multi-locus

## Abstract

**Background:**

Molecular autopsy refers to DNA-based identification of the cause of death. Despite recent attempts to broaden its scope, the term remains typically reserved to sudden unexplained death in young adults. In this study, we aim to showcase the utility of molecular autopsy in defining lethal variants in humans.

**Methods:**

We describe our experience with a cohort of 481 cases in whom the cause of premature death was investigated using DNA from the index or relatives (molecular autopsy by proxy). Molecular autopsy tool was typically exome sequencing although some were investigated using targeted approaches in the earlier stages of the study; these include positional mapping, targeted gene sequencing, chromosomal microarray, and gene panels.

**Results:**

The study includes 449 cases from consanguineous families and 141 lacked family history (simplex). The age range was embryos to 18 years. A likely causal variant (pathogenic/likely pathogenic) was identified in 63.8% (307/481), a much higher yield compared to the general diagnostic yield (43%) from the same population. The predominance of recessive lethal alleles allowed us to implement molecular autopsy by proxy in 55 couples, and the yield was similarly high (63.6%). We also note the occurrence of biallelic lethal forms of typically non-lethal dominant disorders, sometimes representing a novel bona fide biallelic recessive disease trait. Forty-six disease genes with no OMIM phenotype were identified in the course of this study. The presented data support the candidacy of two other previously reported novel disease genes (*FAAH2* and *MSN*). The focus on lethal phenotypes revealed many examples of interesting phenotypic expansion as well as remarkable variability in clinical presentation. Furthermore, important insights into population genetics and variant interpretation are highlighted based on the results.

**Conclusions:**

Molecular autopsy, broadly defined, proved to be a helpful clinical approach that provides unique insights into lethal variants and the clinical annotation of the human genome.

**Supplementary Information:**

The online version contains supplementary material available at 10.1186/s13073-021-00973-0.

## Background

Autopsy is intricately linked to medicine since antiquity and has been fundamental to our understanding of disease mechanisms. Unfortunately, however, there has been a steady decline in autopsy rates due to a combination of factors [1]. Leveraging the power of DNA to reveal the cause of death, Ackerman and colleagues coined the term “molecular autopsy” in 1999 when describing their approach of sequencing *KVLQT1* in a cohort of young adults who died of unexplained drowning [2]. Since then, there has been a sustained growth of the literature on molecular autopsy with evolution of the technology used from targeted variant analysis to Sanger sequencing of one or more genes of interest to next-generation sequencing gene panels to whole-exome sequencing. However, the term remained largely confined to the study of unexplained death among young adults, e.g., a very recent “genomic autopsy” study reported the use of whole-genome sequencing to determine the cause of death in young adults averaging 23 years of age [3]. In 2018, we suggested an expansion of molecular autopsy to also include its application in maternal-fetal medicine (lethal malformation, stillbirths, intrauterine fetal deaths, etc.), which was subsequently endorsed by others [4, 5].

By focusing on the identification of lethal variants, molecular autopsy has the potential to make important clinical and scientific contributions to the field of human genomics. Defining the cause of death at the molecular level provides diagnostic precision, allows accurate genetic counseling, and enables management changes among surviving relatives. Furthermore, molecular autopsy improves the interpretation of the clinical sequencing of the human genome (gene-level and variant-level) by revealing “gene essentiality” [6] and helps expand the spectrum of phenotypic expression of genes. In addition, novel evolutionary insights can be gained by comparing the pattern of lethal variants in humans vs. model organisms [7]. Improved knowledge about lethal variants is also directly relevant to population genomics [8]. To maximize these benefits, we have sought in this study to utilize molecular autopsy broadly defined in a large cohort.

## Methods

### Human subjects

We used a broad case definition of any death before age 18 years with a suspected genetic etiology. The result of the molecular investigation, whether negative, ambiguous, or positive, will have been obtained after death. EDTA blood was obtained from 481 Saudi families (collected during the period 2009 through 2021), and in some instances, we also obtained sodium heparin blood or skin biopsy as needed. Samples were collected only after signing a written informed consent form by the parents. The research was approved by the local IRB (KFSHRC RAC# 2080006, 2121053, 2140016, 2080033, and 2070023). The molecular tools used for the analysis of the families were exome sequencing (*n* = 383), gene panel (*n* = 80), positional mapping and targeted sequencing (*n* = 12), RNA-seq (*n* = 1), WGS (*n* = 1), and chromosomal microarray sequencing for all simplex cases. This heterogeneous testing modality was driven by the availability of certain genetic testing at the time of investigations. The study included 163 previously unpublished families.

### Variant identification and classification

All cases recruited after 2012 were analyzed using exome sequencing (ES). Earlier cases were analyzed using targeted panels relevant to the clinical phenotype as described before [9]; negative cases were then submitted for ES. In a very small number of cases, positional mapping and targeted gene sequencing strategy (*n* = 12), RNA-seq (*n*=1), or whole-genome sequencing (*n* = 1) were used. Candidate variants were selected on the basis of clinical relevance and were classified according to ACMG guidelines [10]. Cases were considered “solved” if a pathogenic or likely pathogenic variant was identified that explains the phenotype, ambiguous outcome refers to variants of uncertain significance (VUS, including variants in novel candidate genes); otherwise, the case was considered “negative.” When DNA from the index is not available, duo ES was pursued in both parents (molecular autopsy by proxy) as described [4]. Chromosomal microarray (CMA) was applied on all simplex cases before subsequent analysis with ES or gene panels was pursued. Local minor allele frequency (MAF) was calculated based on an in-house dataset of 2379 local, ethnically matched exomes.

### Statistical testing

*p* values for statistical analysis of diagnostic yield were obtained using the chi-square test.

### Web resources

The following web resources were used to document different relevant information as follows: Data from Deciphering the Mechanisms of Developmental Disorders (https:/dmdd.org.uk), a program funded by the Wellcome Trust with support from the Francis Crick Institute, is licensed under a Creative Commons Attribution license and was used for the phenotype of *﻿Ehbp111* knockout mice. Genome Aggregation Database https://gnomad.broadinstitute.org [11] was used to check the frequency of identified variants in different populations. Pfam database, http://pfam.xfam.org/ [12], was used to determine protein domains and map the mutated base. Finally, ClinGen, https://clinicalgenome.org [13], criteria were applied to upgrade the classification of some variants by upgrading the gene-disease relationship.

## Results

**Fig. 1:**
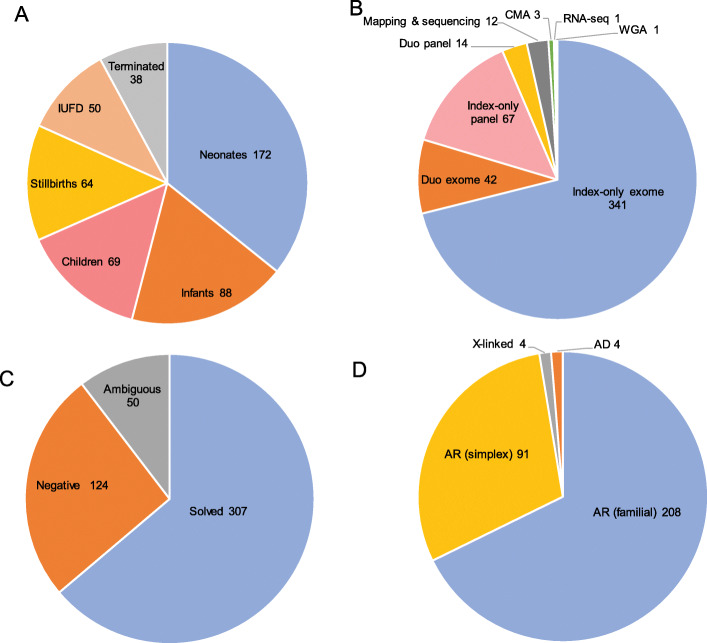
Pie chart summary of the study. **A** Age at death of the study cases. **B** Molecular tools used for the analysis in the study. **C** Molecular outcome of the study. **D** Inheritance pattern of the variants (pathogenic and likely pathogenic) identified in the study

We enrolled 481 index cases in this analysis including 163 previously unpublished cases (Additional file 1: Table S1). The study cases represent a very wide range of phenotypes (see Additional file 2: Fig S1-S9 for representative images) and age at death: intrauterine fetal death (IUFD) (*n* = 50), pregnancy terminations due to lethal malformations (*n* = 38), stillbirths (*n* = 64), neonates (*n* = 172), infants (*n* = 88), and children less than 18-year-old (*n*= 69) (Fig. 1). Consanguinity was documented in the majority (*n* = 449). Positive family history was noted in 340, while the rest were simplex. CMA was performed at baseline for all simplex cases. Those with negative CMA results as well as all familial cases underwent the following testing strategies: index-only ES (*n* = 341), duo ES on the parents (*n* = 42), index-only panel (*n* = 67), duo panel on the parents (*n* = 14), autozygosity mapping and targeted sequencing (*n* = 12), whole-genome sequencing (*n* = 1), and RNA-seq (*n* = 1) (Fig. 1).

### Higher yield compared to regular genetic diagnosis

A likely causal variant was identified in 63.8% (*n* = 307/481, solved), and in 50 cases (10.3%), the candidate was a variant of uncertain significance (ambiguous), while no candidate variant was identified in 124 (25.7%, negative) (Fig. 1 and Additional file 1: Table S1). The percentage of solved cases is significantly higher than what we published for general diagnostic exomes based on >2200 families from the same population (307/481 (63.8%) vs. 960/2219 (43.2%), *p* < 0.0001) [14]. This may be in part due to a higher percentage of loss of function (LOF) variants in this cohort compared to the general diagnostic cohort (187/307 (60.9%) vs. 470/961 (48.9%), *p* = 0.0006). Indeed, the percentage of negative cases is comparable between the two cohorts (124/481 (25.7%) vs. 602/2219 (27%), *p* = 0.28), which suggests that the higher solve rate is primarily driven by a larger P/LP to VUS ratio. Of note, the yield was higher among consanguineous families (294/449 (65.4%) vs. 13/32 (40%)) and in certain phenotypic categories, e.g., 78% among terminated pregnancies (Additional file 1: Table S1 and Additional file 3: Table S2).

### Predominance of autosomal recessive trait-associated biallelic variants

Although CMA was performed on all simplex cases (*n* = 141), a causal variant was identified in the form of chromosomal deletion in only three cases (14DG1134, 16DG1465, and 15DG0267); the latter is a case of holoprosencephaly linked to a large homozygous deletion that encompasses *ZIC2* and *ZIC5* (Additional file 1: Table S1, Additional file 2: Figure S3 A-B). On the other hand, exome and panel sequencing revealed dominant (*de novo*) variants (*ATP1A3* and *NALCN*) in three cases and X-linked variants (*ATP11C*, *PDHA*, *MSN*, and *FAAH2*) in four cases. In comparison, autosomal recessive variants accounted for the overwhelming majority of solved cases (96.7%, 297/307). Importantly, 30.9% (92/297) of the solved cases with recessive variants lacked family history, i.e., simplex (Fig. 1), which highlights the importance of autosomal recessive variants in consanguineous populations even in the setting of negative family history.

### Discovery and support of disease gene candidates

**Fig. 2:**
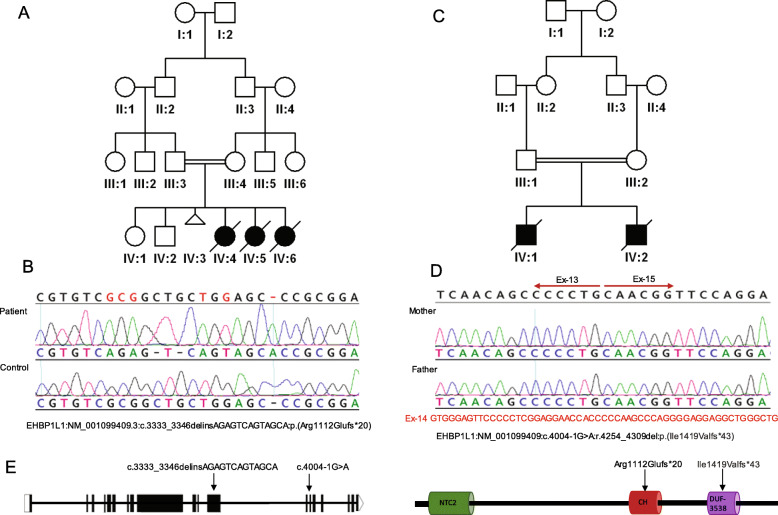
Identification of *EHBP1L1*-related lethal phenotype. **A**, **B** Pedigree of 14DG1037 and sequence chromatogram of *EHBP1L1*:NM_001099409.3:c.3333_3346delinsAGAGTCAGTAGCA variant. **C**, **D** Pedigree of 18DG0247 and RT-PCR of the variant *EHBP1L1*:NM_001099409:c.4004-1G>A) confirming aberrant splicing with skipping of exon-14. **E** Sketch for EHBP1L1 transcript and protein, arrows denote mutated bases and residues

Compelling variants were identified in 46 genes with no established link to Mendelian phenotypes at the time of analysis (Additional file 3: Table S3). Of particular interest is *EHBP1L1* because it was independently identified in two families with non-immune hydrops fetalis (NIHF) resulting in recurrent fetal loss. The first case 14DG1037 was found to be homozygous for *EHBP1L1*: NM_001099409.3:c.3333_3346delinsAGAGTCAGTAGCA:p.(Arg1112Glufs*20) while the second family was analyzed by the duo exome of the parents [14], who were found to be heterozygous for the splice variant NM_001099409:c.4004-1G>A that resulted in skipping of exon 14 and the creation of an aberrant transcript (Fig. 2). EHBP1L1 was identified as a novel binding protein of Rab8, a highly conserved Rab guanosine triphosphatase (GTPase) that plays an essential role in exocytosis toward the polarized plasma membrane [15]. Indeed, deficiency of EHBP1L1, like that of Rab8, results in the lysosomal accumulation of apical but not basolateral membrane proteins. Furthermore, *Ehbp1l1−/−* die within 24h of birth although very few details are described about their pathology apart from abnormal intestinal microvilli [15]. Another line described by DMDD (https://dmdd.org.uk/mutants/Ehbp1l1) showed that *Ehbp1l1*^*−/−*^ is lethal with subcutaneous edema, perimembraneous ventricular septic defect, and thin myocardium.

**Fig. 3:**
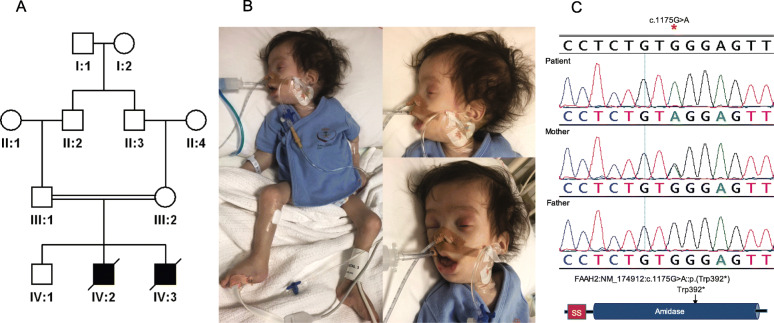
Identification of *FAAH2*-related lethal phenotype. **A** Pedigree of 18DG0989 (died during the neonatal period). **B** Images of the patient with facial dysmorphism, significant hypotonia, and mechanical ventilator dependence. **C** Sanger sequence of *FAAH2*:NM_174912:c.1175G>A:p.(Trp392*) and sketch of FAAH2 protein domains

**Fig. 4:**
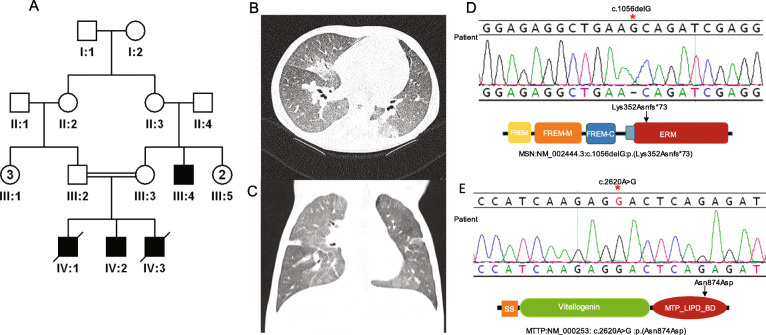
Identification of *MSN*-related lethal phenotype. **A** Pedigree of 16DG0125 (two siblings died during childhood). **B**, **C** CT scan showing widespread ground glass change with no significant interstitial thickening. **D**, **E** Sequence chromatogram of *MSN:*NM_002444.3:c.1056delG, *MTTP*:NM_000253: c.2620A>G variants, and sketches of MSN and MTTP protein domains; arrows denote mutated residues

We also provide supporting evidence of the previously published candidacy of two other genes. Patient 18DG0989 presented with a classical Zellweger syndrome phenotype including global developmental delay, seizure disorder, severe hypotonia, failure to thrive, adrenal insufficiency and elevated very long-chain fatty acids and liver enzymes (Fig. 3 and Additional file 3: Table S4). He was found to be hemizygous for *FAAH2*:NM_174912.3:c.1175G>A:p.(Trp392*) inherited from the normal mother. *FAAH2* was first proposed as a novel candidate disease-related gene by Sirrs et al. who described a male with a neurodevelopmental disorder characterized by neonatal hypotonia, epilepsy, autism, and white matter abnormalities [16]. Additional cases were reported by others albeit with few clinical details [17, 18]. Again, there remains no phenotypic listing for *FAAH2* in OMIM. A second example is patient 16DG0125 who died as a young child of severe respiratory infections, complicated by pulmonary hypertension and progressive occlusion of pulmonary veins although no underlying etiology was identified (Fig. 4). We identified a hemizygous truncating variant in *MSN* (NM_001931.3:c.975G>A:p.(Lys352Asnfs*73)), which was proposed as a candidate gene for immunodeficiency based on a single publication [19]. Interestingly, the same maternally inherited variant was also found in a brother and a maternal cousin who are both still alive and healthy, which is consistent with the highly variable age of onset of *MSN*-related immunodeficiency [19].

### Phenotypic expansion

As expected for a cohort with lethal phenotypes, we encountered instances of lethal presentations of genes typically linked to much milder phenotypes. Perhaps the most striking is the family with *FZD6* variant alleles. Although the only phenotype linked to this gene in OMIM is a nail-limited abnormality, we have previously described a family with NIHF and proposed *FZD6* as the likely candidate [20]. The second family we describe here is one of three families with *FZD6*-related NIHF that will be published elsewhere (Sheppard et al., manuscript in preparation). Remarkably, this family has multiple members with the classical *FZD6*-related nail disorder with history of NIHF during pregnancy, which suggests that *FZD6*-related NIHF, like *THSD1*-related NIHF [4, 20], can be compatible with long-term survival if mortality in the newborn period is avoided. Additional examples include case 09DG01414 with a homozygous truncating variant in *TMPRSS15* (NM_002772.2:c.2808_2809insATCA p.(Ser937Ilefs*4)) who died in infancy, an unusual presentation of enterokinase deficiency. Similarly, case 21DG0001 with a homozygous truncating variant in *TNNT3* (NM_006757:c.723-2A>G) presented with severe non-immune hydrops fetalis and neonatal death (Additional file 2: Fig. S5), which have not been reported to date in *TNNT3*-related myopathy. In the same context, duo ES for parents of a deceased neonate (19DG0461) with a history of fetal akinesia and polyhydramnios revealed the shared carrier status of a splice variant in *TTN* (NM_001267550.2:c.28462+1G>T), which likely explains the phenotype of the index patient in the homozygous state as reported by others [21–23].

We also highlight case 17DG0032 whose lethal Neu-Laxova syndrome-like presentation represents a phenotypic expansion compared to the four patients reported to date with the lethal *LARS2*-related HLSA (hydrops, lactic acidosis, and sideroblastic anemia) syndrome (Altawil et al., manuscript in preparation and [24, 25]).

### Variants in “dominant” genes that are lethal in the biallelic state

We observed two classes of lethal variants in genes that are typically linked to dominant phenotypes only. First, there are recessive variants with no apparent clinical consequences in the carrier parents but result in a lethal phenotype in the offspring. Patient 16DG1276 with a homozygous *LZTR1* variant and NIHF (Additional file 2: Fig.S6) was proposed as the first instance of a truly recessive inheritance of this Noonan syndrome-linked gene, which was later confirmed by others [4, 26]. Similarly, although *MYH11* has been tentatively linked to adult-onset aortic aneurysm, we have one case (17DG1094) with a homozygous truncating variant (NM_022844:exon9:c.1033+1G>A) and had a distinct fetal presentation (Additional file 2: Fig.S7) consistent with several reported megacystis-microcolon syndrome cases caused by biallelic *MYH11* variants [14, 27]. Currently, aortic aneurysm is the only phenotype listed by OMIM under *MYH11*. Similarly, homozygous variants in *FBN2* and *KRIT1*, linked to autosomal dominant congenital contractural arachnodactyly and cerebral cavernous malformations 1, respectively, inherited from healthy heterozygous parents were identified in patients with arthrogryposis and NIHF, respectively. Furthermore, a previously reported likely pathogenic *BMPR1A* variant was found to be shared by normal consanguineous parents who presented for prenatal counseling regarding recurrent skeletal dysplasia and IUFD (20DG1384). We propose this to be a novel recessive phenotype of *BMPR1A*, a gene hitherto linked to autosomal dominant intestinal polyposis, which would be in line with our previous finding of a novel recessive phenotype of *APC* comprising severe bone malformations [28, 29].

The second class is dominant variants that were rendered homozygous by the union of two affected heterozygous parents, i.e., semidominant inheritance. For example, the pregnancy of 16DG0393 was terminated because of the identification of a homozygous *PKD1* pathogenic variant resulting in severe polycystic kidneys and posterior fossa abnormality consistent with the published literature, while the consanguineous asymptomatic parents were found in retrospect to have autosomal dominant polycystic kidney disease [30, 31]. Similarly, patient F8317 died of severe skeletal dysplasia caused by a known achondroplasia-related *FGFR3* variant inherited from both affected parents (Additional file 2: Fig. S8).

### Extreme phenotypic variability of some variants depending on the genotype

Family 15DG2154 presented with the lethal microcephaly-micromelia syndrome in multiple pregnancies, which was mapped to a severe splicing variant in *DONSON*, the same variant that when inherited in trans with milder variants results in a much milder Seckel syndrome as described previously [32] (a range of *DONSON*-related phenotypes has also been described [33]). A similar scenario is found in 20DG0807 with a homozygous variant in *CHAT* resulting in IUFD, while compound heterozygosity for the same variant with a milder variant was reported to result in a viable congenital myasthenic phenotype [34].

### Implications for MAF interpretation

In patient 15DG0936 with a lethal form of hepatic ciliopathy, we initially dismissed the candidate variant *TTC26*:NM_001144923.2:c.4-1G>C because of its relatively high local population frequency (MAF:0.002); however, it was proven causal on RT-PCR as previously described [35]. This frequency should predict a more common contribution of this founder variant to our molecular autopsy cohort than observed. This discrepancy may be explained by the success of liver transplant in preventing death among affected children. This founder variant is one of 100 founder variants that explained nearly half of the solved cases 161/307 (52.4%). Interestingly, most of the founder variants (67% (67/100)) are unique to the local population, while the rest were encountered in other world populations (Additional file 3: Table S5).

### The potential of other “omics” in molecular autopsy

**Fig. 5:**
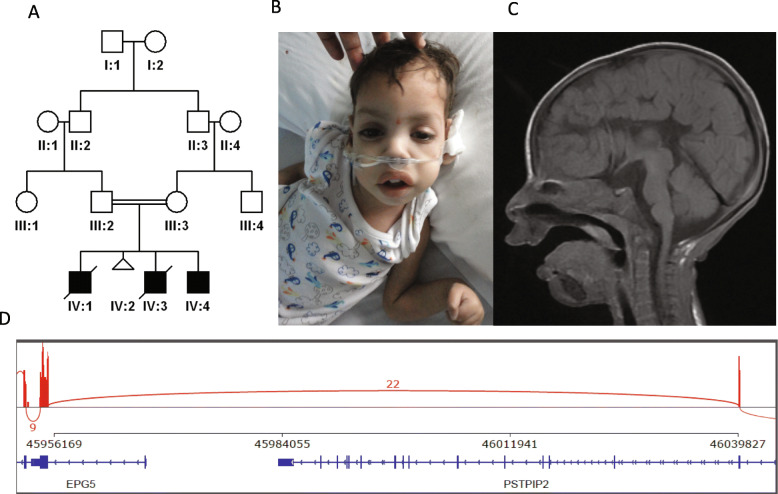
RNA-seq identifies *EPG5*-related lethal phenotype. **A**, Pedigree of 16DG1465 with deletion of *EPG5* exon-1 (two siblings died during infancy). **B** Image of the patient with facial dysmorphism. **C** MRI showing absent corpus callosum. **D** A sashimi plot showing the base-level density of reads mapped to a genomic region surrounding the ~70K deletion site. The *x*-axis represents the genomic coordinate in hg38. The *y*-axis represents per-base read counts. On the bottom, the boxes are annotated exons, the horizontal lines are introns, and the left-facing arrowheads indicate the negative strand. Arcs connecting exons represent splice junction reads. The plot shows that no reads are mapped to exon 1 of *EPG5* and that 22 split-reads are mapped to an aberrant splicing junction between exon 2 of *PSTPIP2* and exon 2 of *EPG5* that are ~84Kbp apart

In addition to the overrepresentation of conspicuous LOF variants in our cohort, cryptic LOF variants were also observed. One example is patient 13DG1520 who died as a neonate and initially had negative exome results. However, reanalysis revealed a homozygous synonymous variant in *DLAT* (NM_001931.3:c.975G>A:p.(=)) [36]. RT-PCR confirmed the splicing nature of this variant (r.788_975del: p.(Phe264Glyfs*32), which was the last nucleotide of exon 6 and was, therefore, reclassified as LOF. Interestingly, this case also represents a phenotypic expansion of *DLAT*-related pyruvate dehydrogenase deficiency. A more challenging case was family 11DG1647 that lost several children with severe neonatal lactic acidosis, which could not be solved by exome sequencing. Positional mapping allowed us to prioritize a variant that had been dismissed because it was synonymous in an apparently non-splicing position (*BCS1L*:NM_001079866:exon3:c.441C>T:p.F147F). RT-PCR using parental samples, however, revealed that this is a strong exonic splicing variant and confirmed its LOF nature (Additional file 2: Fig. S9). Although these cases were solved by targeted RT-PCR, they highlight the potential role of transcriptomics to supplement exome sequencing at least in select cases. Indeed, in family 16DG1465 that lost four children with Hirschsprung disease, gastroesophageal reflux disease, coarse facial features, severe global developmental delay, agenesis of the corpus callosum, failure to thrive, and cataract, exome sequencing was negative but RNA-seq as described before [36] highlighted an abnormal profile of *EPG5* and subsequent analysis revealed a homozygous deletion of exon 1 (Fig. 5).

### Multi-locus pathogenic variation: families with multiple lethal phenotypes

In the above described patient 16DG0125 with a hemizygous truncating variant in *MSN*, we also identified a homozygous variant in *MTTP* (NM_000253:c.A2620G:p.Asn874Asp) that may have contributed to his demise due to severe diarrhea (Fig. 4). On the other hand, we report one family that lost two pregnancies due to NIHF, and a different homozygous variant was identified in each: 13DG0259 was homozygous for *FCRL4:* NM_031282:c.847+1G>A while 15DG0933 was homozygous for *SLC17A5*:NM_012434:c.1111+1G>A. This has important implications on the utility of segregation analysis of lethal variants in inbred families and a reminder of the residual risk for additional autosomal recessive traits in consanguineous families that present for counseling regarding one autosomal recessive trait [37].

## Discussion

In this cohort, we describe our experience with lethal variants identified by molecular autopsy in young patients with suspected genetic etiology. As expected for a population enriched for consanguinity [38, 39], the overwhelming majority of variants were autosomal recessive, even when positive family history was lacking. In comparison, only 5 out of 9 variants identified by Quinlan-Jones et al. in their smaller study with a narrower definition of molecular autopsy (lethal malformations during pregnancy) were recessive [5]. Similarly, the study by Stanley et al. on genetic causes of stillbirths observed that only 20% of the causal variants were recessive [40]. Thus, the two ways in which lethal variants can circumvent the reproduction barrier because of their low reproductive fitness, i.e., to arise de novo (dominant) or to pass to the next generation in the monoallelic state (recessive), can be observed in action albeit to a variable degree depending on the population structure, e.g., inbred vs. outbred [41].

Despite the heterogeneous nature of this cohort, the lethal nature of the causal variants represents a common denominator that we think contributes to the high diagnostic rate. In other words, it is likely that lethal variants are more likely to belong to classes that are more readily identified and classified as pathogenic or likely pathogenic, and this is supported by our finding that this cohort is enriched for LOF variants (see below). Consistent with this notion, a higher than average diagnostic rate has also been reported in ES sequencing of children with severe phenotypes in intensive care units [42]. The detailed listing of phenotypes in our cohort should allow comparison to other studies that focused on specific phenotypes. For example, the yield among pregnancy terminations for fetal malformations was 78% in this cohort (Additional file 3: Table S2 ) compared to 20% (74% if ambiguous cases were also included) by others [43].

We have taken advantage of this predominance of autosomal recessive variants to identify the likely cause of death when no DNA sample from the deceased individual is available by studying the presumptive carrier parents. This “molecular autopsy by proxy” approach has proven helpful in the majority of families who could now receive accurate genetic counseling and make informed reproductive choices. Unlike the de novo paradigm, the recurrence risk in the autosomal recessive paradigm is high, which underscores the clinical utility in making a precise molecular diagnosis that can be the basis for such preventative measures as cascade carrier testing, prenatal diagnosis, and preimplantation genetic testing [44]. Although our focus in molecular autopsy by proxy was on recessive variants, we should note the important contribution of balanced chromosomal rearrangements in asymptomatic parents to lethal phenotypes among the unbalanced offspring. Dong et al. found that 11.7% of couples with recurrent miscarriage harbor balanced rearrangements many of which could only be identified using genome sequencing [45]. This suggests that future studies into molecular autopsy by proxy should consider screening parents for both SNVs and CNVs (including balanced rearrangements) to maximize the yield.

The enrichment for LOF in lethal variants has been noted by others [40]. This may have contributed to the high yield of the molecular autopsy by enabling more variants to be classified as pathogenic or likely pathogenic. Several points are worth highlighting in this regard. First, not all LOF are readily identifiable, especially transcript-deleterious variants that masquerade as benign synonymous variants in non-canonical exonic splice sites. Although positional mapping has proven very helpful in highlighting these variants [46], we acknowledge that this is not always feasible. On the other hand, RNA-seq has been shown to effectively identify and properly classify such variants [36], so it is likely to have a prominent role in future molecular autopsy studies as shown in the example described here. Second, there is a growing appreciation of genetic disorders that are only caused by a combination of mild and severe variants in trans because homozygosity for the mild variants may be asymptomatic while homozygosity for the severe variant is embryonically lethal. Molecular autopsy in consanguineous populations is an ideal setup for the latter class of LOF as shown in the example involving the *DONSON* variant. This is a reminder that the clinical interpretation of a variant must take its genotype into consideration because a variant may be lethal in one context but not others. Third, caution must be exercised when interpreting LOF variants in genes linked to dominant conditions when these are encountered in asymptomatic individuals. The tendency to rule these as examples of non-penetrance should be balanced by the possibility that these may represent bona fide autosomal recessive inheritance, which has a very different recurrence risk estimate [47]. Lastly, the use of population frequency to interpret the pathogenicity of an apparently LOF variant can be deceiving unless a database of lethal variants is established from the same population.

“Embryonic lethal genes” is a term used commonly in model organisms and has been useful in highlighting genes that are likely to be disease-related in humans as shown in the mouse and fruitfly [48, 49]. However, this term overlooks the unlimited “allelic series” of the corresponding genes in humans and the remarkable phenotypic diversity thereof. This is why it is difficult to have an accurate estimate of the percentage of “embryonic lethal genes” in humans in comparison to the available estimates from model organisms. Even in this molecular autopsy cohort where the variants are by definition lethal, death during fetal development was only observed in a subset of cases. *PHGDH* is a case in point. *Phgdh*−/− mice die during embryonic development while in humans variants in this gene can result in phenotypes ranging from stillbirths due to Neu-Laxova syndrome to the much milder serine deficiency-related neurodevelopmental disorder [50, 51]. It should also be noted that embryonic lethality may even precede biochemical pregnancy as demonstrated in *TLE6* that has been linked to the earliest possible form of embryonic lethality, involving failure of zygote cleavage, and other variants that have been shown to cause embryonic lethality that can only be identified through IVF clinics [52–54].

Delineating the severe end of the phenotypic expression of Mendelian genes through a broadly defined molecular autopsy approach such as the one implemented in this work can contribute significantly to the clinical annotation of the human genome both at the gene and variant levels. One particularly interesting area of research in population genomics is inferring recessive lethal variants by calculating their depletion in the control population in the biallelic state. The short-read sequencing technology that is typically used in generating available variant databases precludes phasing of most of the rare variants such that depletion of homozygotes is the only practical alternative. However, as the frequency of a variant decreases, the population size required to confidently conclude its depletion in the homozygous state becomes exponentially and impractically larger except in consanguineous populations. Indeed, we note that 36.7% (132/359) of the lethal variants described here exist in gnomAD albeit at such low frequencies that it could not be concluded if they are lethal in the recessive state and yet they were identified in our population despite a comparably low MAF. The one exception (*TTC26:*NM_001144923.2:c.4-1G>C) where the local MAF was high and did not reflect a correspondingly strong representation in the molecular autopsy cohort is a reminder that control databases from a given population afford a limited interpretation power unless they are supplemented by efforts to catalog lethal variants from the same population. It is hoped that this and future molecular autopsy from other populations will help toward that goal.

## Conclusions

We conclude that molecular autopsy is a powerful tool to reveal the cause of death when applied correctly. Novel clinical and biological insights can be gained from this approach, the clinical utility of which, especially when broadly defined, is becoming increasingly clear. In the future, it is likely that molecular autopsy will involve other omics in addition to genome sequencing. The value of molecular autopsy in defining lethal variants in humans on the clinical annotation of the human genome cannot be overstated.

## Supplementary Information

**Additional file 1: **Supplementary Table S1. **Table S1: Details of clinical and molecular findings of the study cases.** No OMIM phenotype*: Phenotype is not listed in OMIM. Likely pathogenic*: Upgraded to likely pathogenic using ClinGen criteria. Negative*: A case with liver cirrhosis and F7 deficiency, the cause of liver cirrhosis is not identified. CGM: local ES database. NA: Unsolved cases. a: Novel gene or variant to be published separately.

**Additional file 2:.** Supplementary Figures S1-S9

**Additional file 3: **Supplementary Tables S2-S5. **Table S2: Level of very long chain fatty acids in patient 18DG0989.** Values with asterisk are elevated. **Table S3: Breakdown of genes with no OMIM phenotype.** No OMIM phenotype*: Case phenotype is not listed in OMIM. **Table S4: Breakdown of the diagnostic yield by phenotypic outcome and consanguinity status.** Genes in bold are mutated in more than one family. **Table S5: Founder variants in this study and their frequency in Saudi and other populations.** CGM: local ES database.

## Data Availability

All variants’ data has been deposited to ClinVar and that ClinVar accession IDs are provided in Additional file 1: Supplementary Table S1. RNA-seq data relevant to the 16DG1465 case have been deposited in NCBI Sequence Read Archive under bioproject ID PRJNA761889 (http://www.ncbi.nlm.nih.gov/bioproject/761889) [55]. Due to local IRB (King Faisal Specialist Hospital and Research Center Research Advisory Council) regulations to protect the privacy of human subjects, individual-level raw data cannot be deposited in public databases.
