# Supplementary material for: Lethal variants in humans: lessons learned from a large molecular autopsy cohort
Source: Genome Med. 2021 Oct 13;13:161. doi: 10.1186/s13073-021-00973-0 (PMC8511862; doi:10.1186/s13073-021-00973-0)
Supplement: Supplementary file 2 — Additional file 2:. Supplementary Figures S1-S9 [file 13073_2021_973_MOESM2_ESM.pdf]

Figure S1

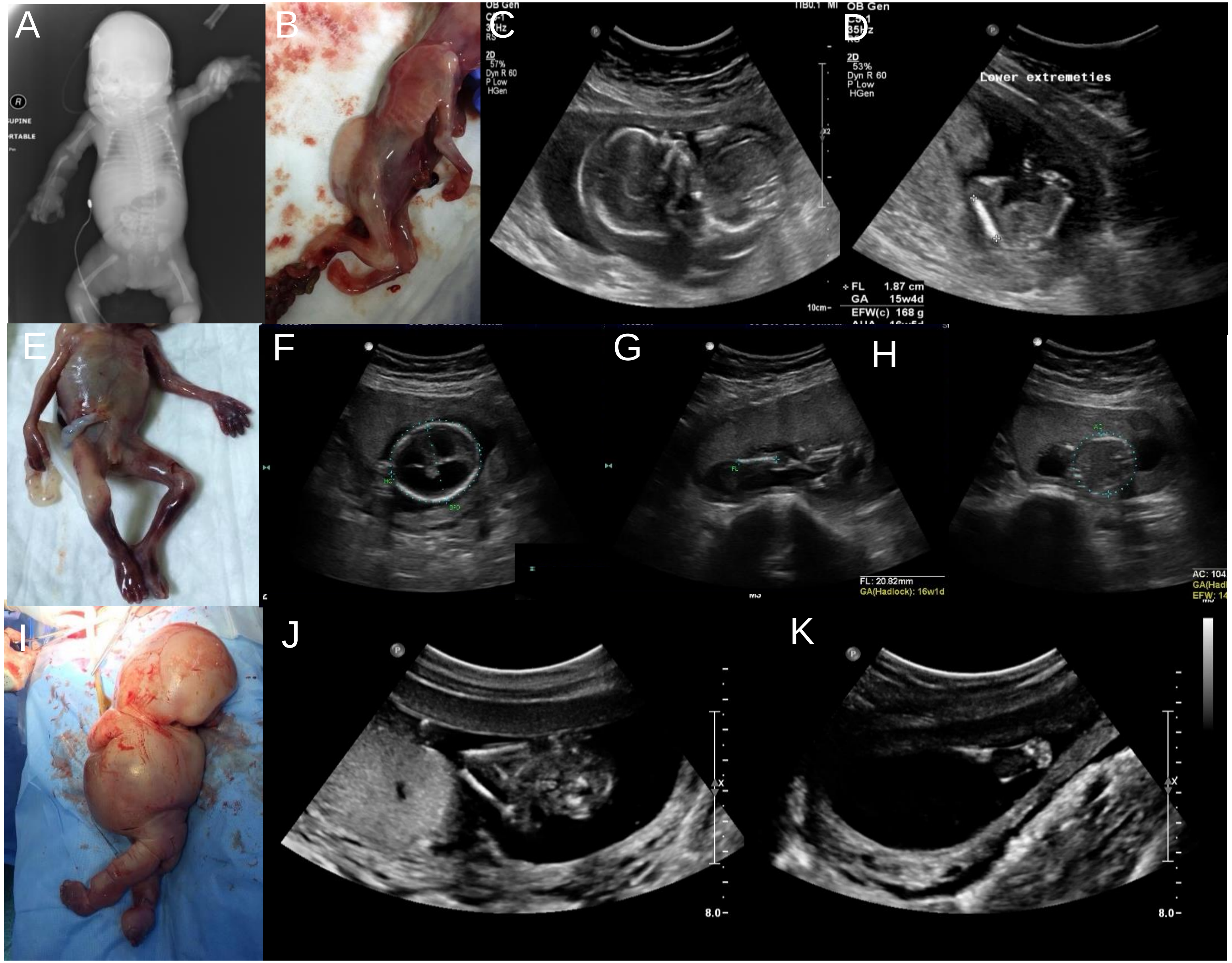
A
B
C
D
Lower extremeties
FL 1.87 cm
GA 15w4d
EFW(c) 168 g
E
F
G
H
FL: 20.82mm
GA(Hadlock): 16w1d
I
J
K
8.0
8.0

Figure S2

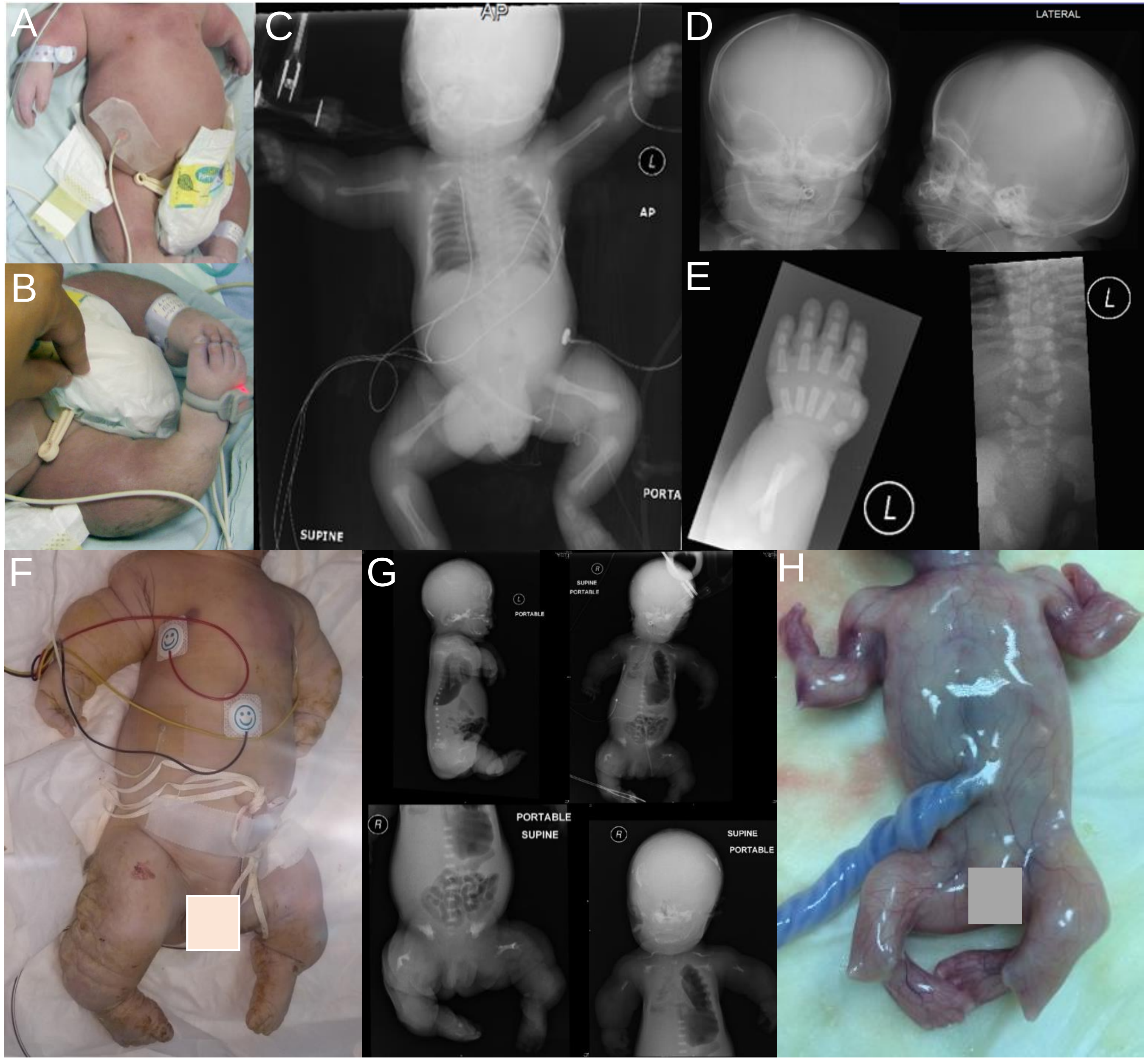

A
B
C
AP
L
AP
SUPINE
PORTA
D
LATERAL
E
L
L
F
G
PORTABLE
SUPINE
PORTABLE
PORTABLE
SUPINE
SUPINE
PORTABLE
H

Figure S3

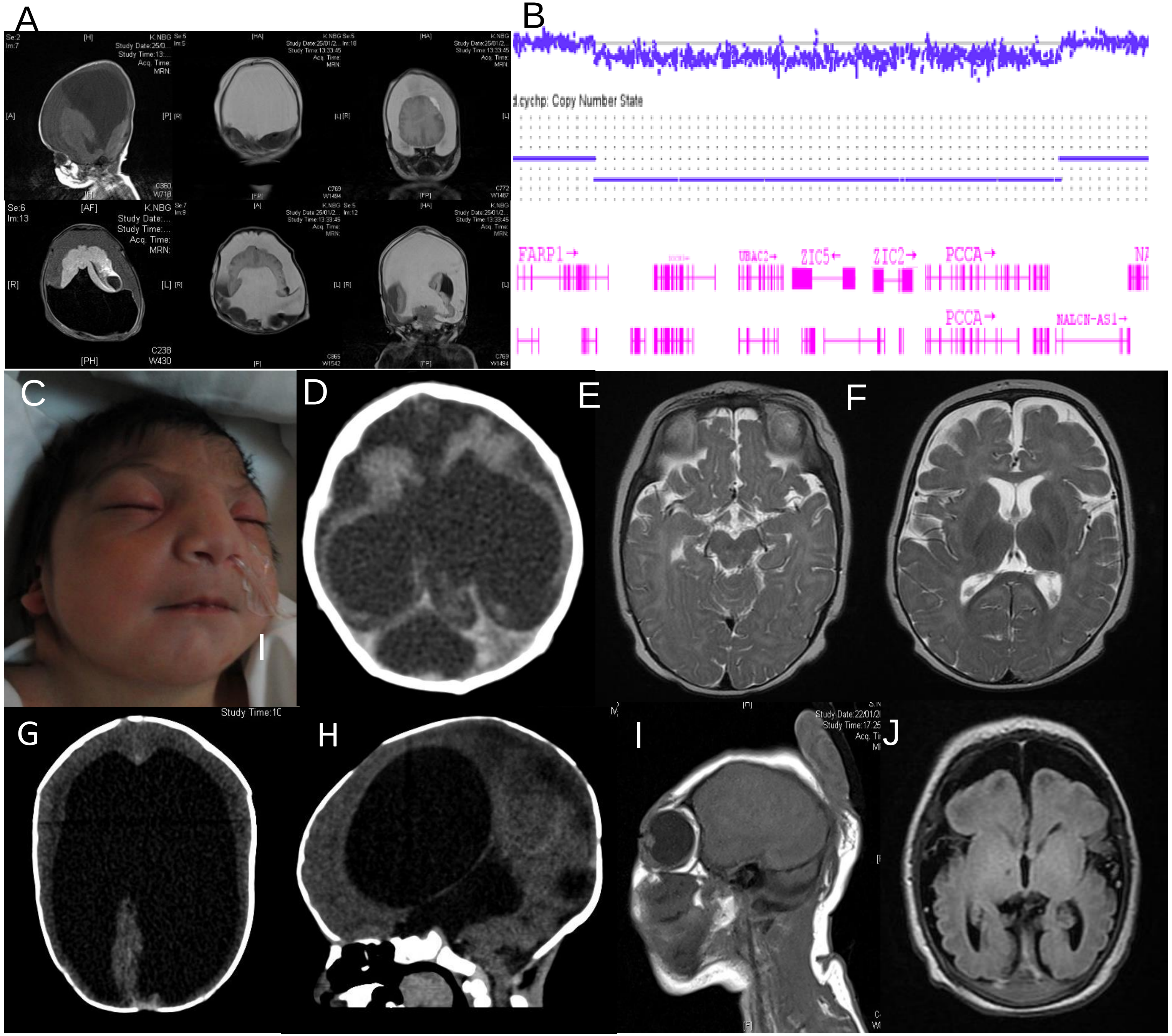
A
B
d.cychp: Copy Number State
FARP1→
UBAC2→
ZIC5←
ZIC2→
PCCA→
PCCA→
NALCN-AS1→
C
D
E
F
G
H
I
J

Figure S4

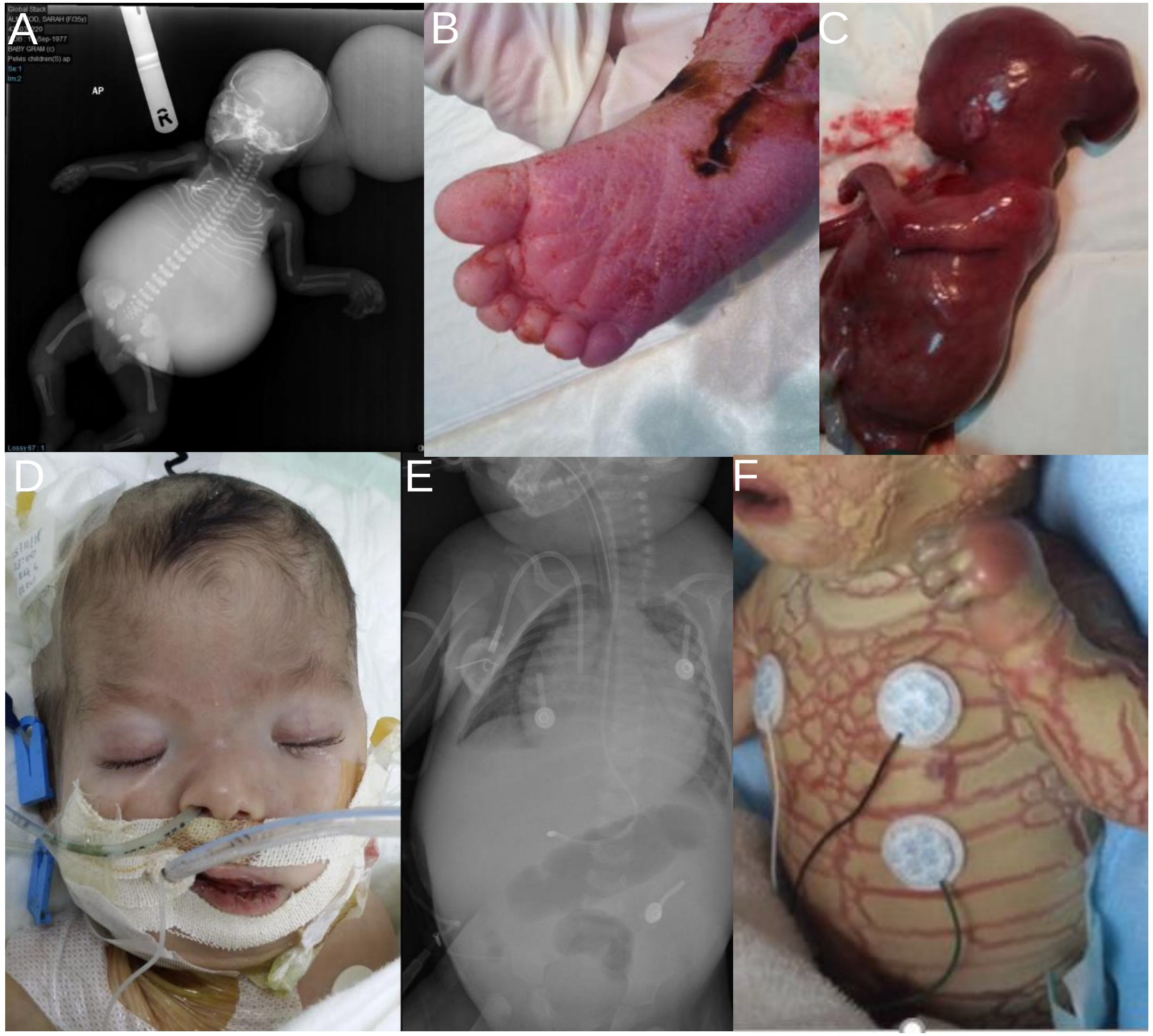

Figure S5

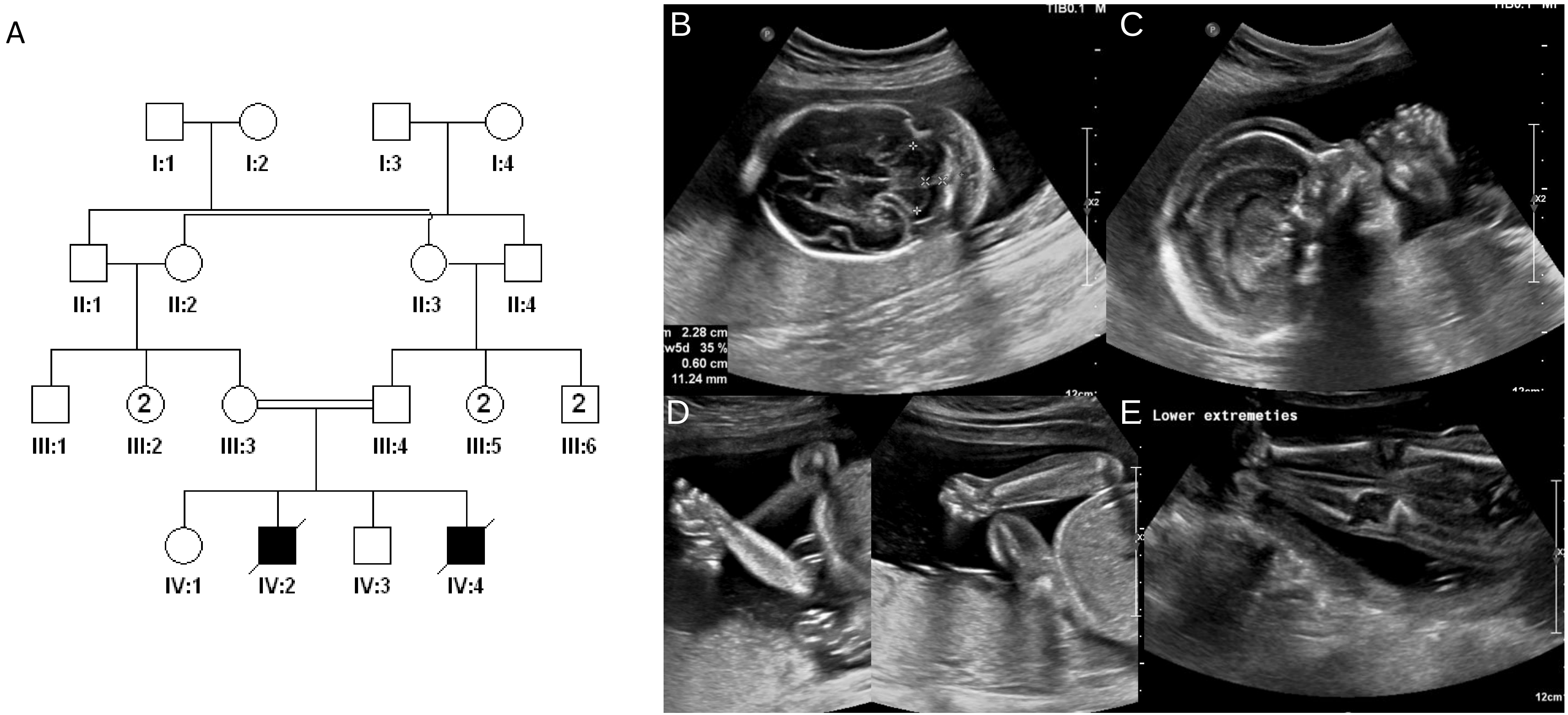
A
I:1
I:2
I:3
I:4
II:1
II:2
II:3
II:4
III:1
III:2
III:3
III:4
III:5
III:6
IV:1
IV:2
IV:3
IV:4
B
2.28 cm
35 %
0.60 cm
11.24 mm
C
D
E
Lower extremeties
12cm

Figure S6

A

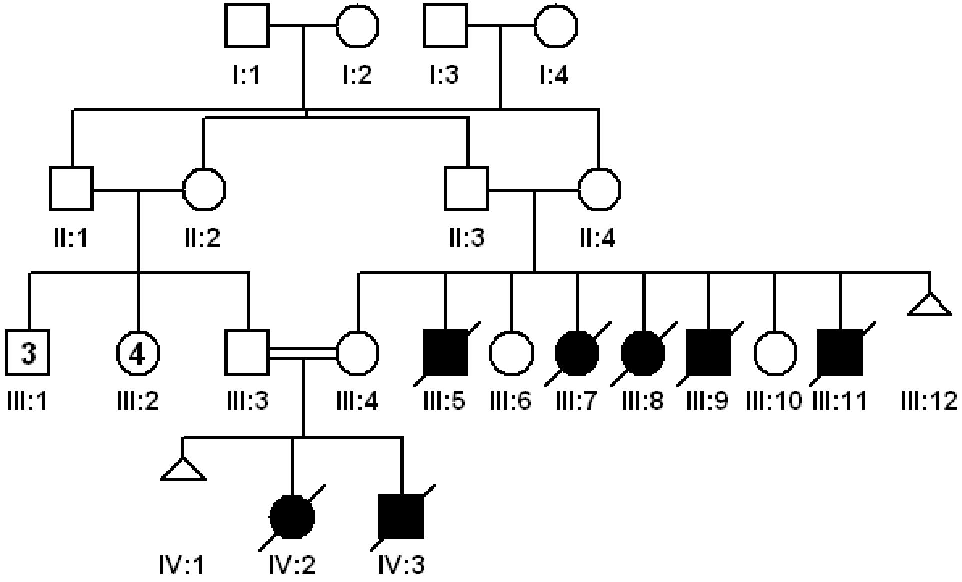
I:1
I:2
I:3
I:4
II:1
II:2
II:3
II:4
3
4
III:1
III:2
III:3
III:4
III:5
III:6
III:7
III:8
III:9
III:10
III:11
III:12
IV:1
IV:2
IV:3

B

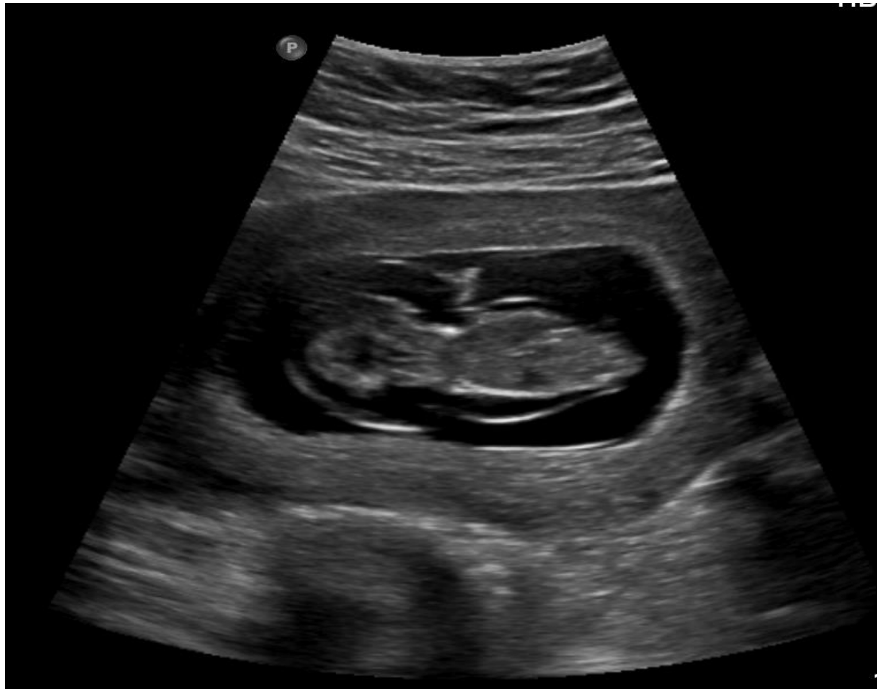

Figure S7

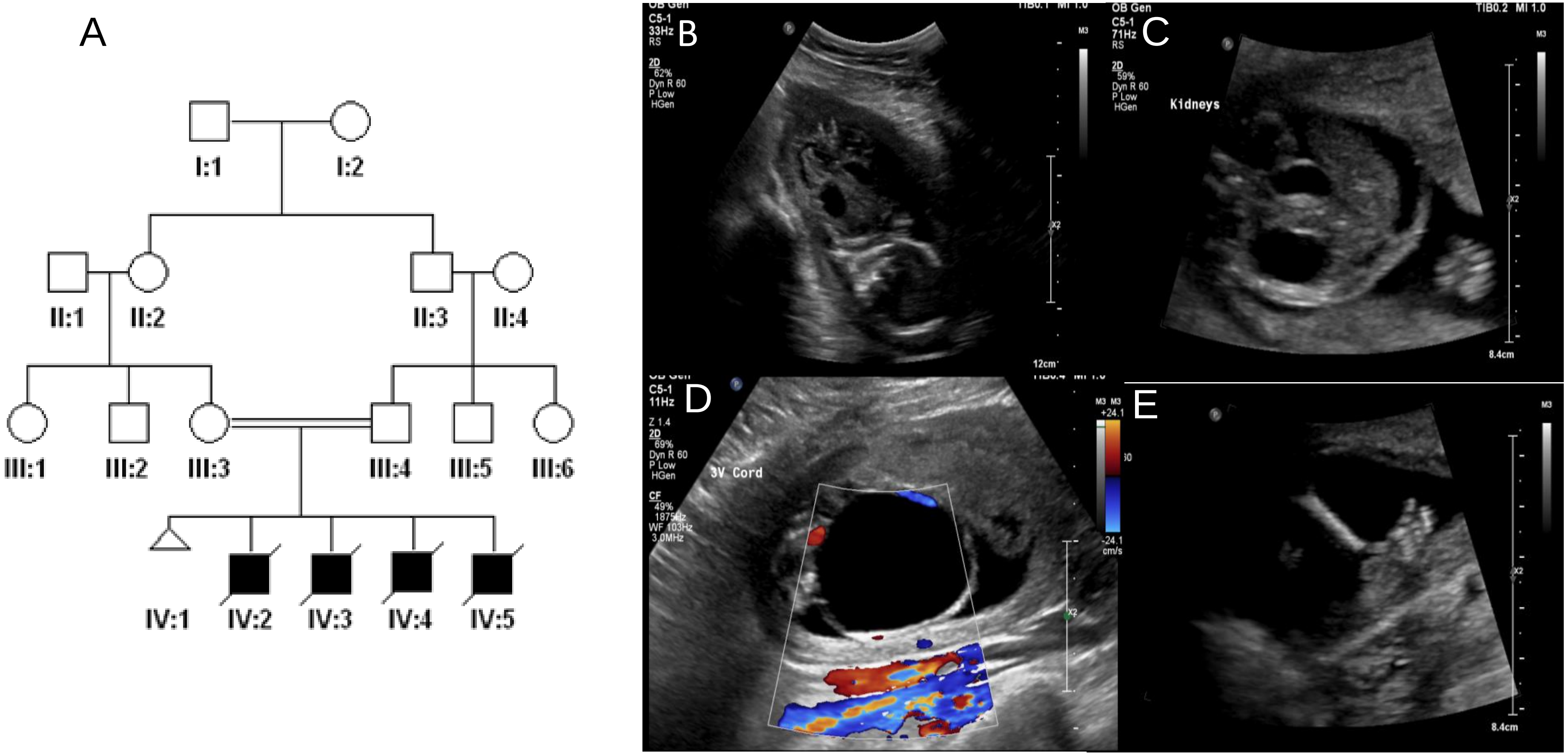
A
I:1
I:2
II:1
II:2
II:3
II:4
III:1
III:2
III:3
III:4
III:5
III:6
IV:1
IV:2
IV:3
IV:4
IV:5
B
C
Kidneys
D
3V Cord
E
12cm
8.4cm
8.4cm

Figure S8

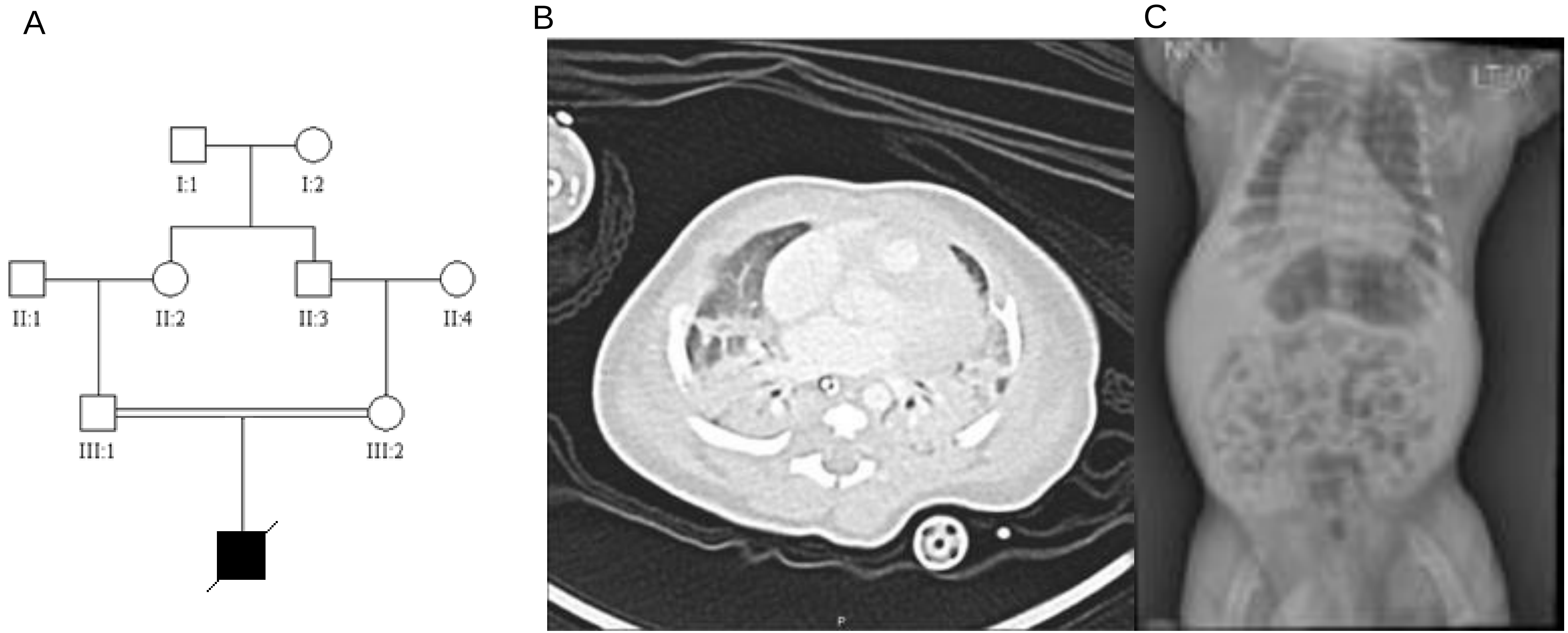

Figure S9

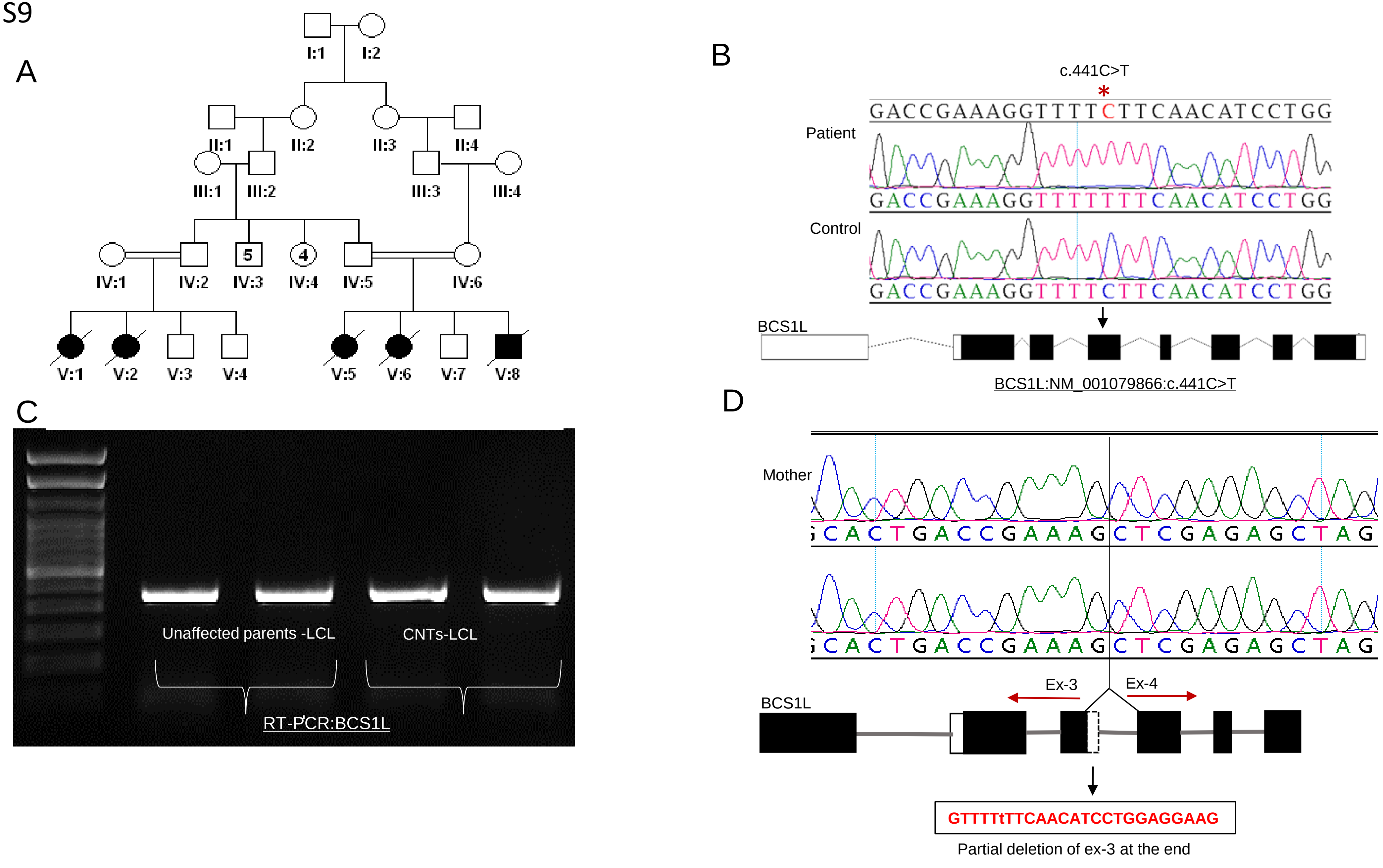

BCS1L:NM_001079866:c.441C>T:r.436_460delGTTTTCTTCAACATCCTGGAGGAAG:p.(Val146Leufs*4)

Supplementary Figures

Figure S1: Representative images of cases with NIHF and death at different stages:

A- X-ray of 09DG01201 (*PIGC*:NM_002642.4:c.659T>C:p.(Leu220Pro) homozygous) with ascites, and short limbs (died during the neonatal period).

B-D) Antenatal ultrasound of 16DG1603 (*NEB*:NM_001164507.2:c.20974delA:p.(Val6993Serfs*8) homozygous) showing scalp edema, nuchal edema, and short long bones (IUFD).

E-H) Antenatal ultrasound of 15DG0174 (*FKTN:* NM_006731.2:c.78C>G:p.(Tyr26*) homozygous) showing brain malformations with dilated 3rd and lateral ventricles, absent choroid plexus dilated posterior fossa with hypoplastic cerebellum and signs of hydrops fetalis (terminated pregnancy).

I) Clinical photograph of 15DG0986 (*MRPS16*:NM_016065.4:c.331C>T:p.(Arg111*) homozygous) showing cystic hygroma, pleural effusion, very short long bones and ascites (stillbirth).

J-K) Antenatal ultrasound of 15DG1840 (*POLG*:NM_001126131.2:c.2606G>A

:p.(Arg869Gln) homozygous) at 17 weeks showing fixed flexion of upper limbs, talipes, and signs of hydrops (IUFD).

Figure-S2: Representative images of cases with skeletal malformations and neonatal death.

A-E) Clinical and radiographic images of 13DG2208 (*ROR2*:NM_004560.2:c.1970G>A:p.(Arg657His) homozygous) showing shortening of upper and lower limbs, macrocephaly, and scoliosis.

F-G) Clinical and radiographic images of 15DG1287 (*ALPL*: NM_001177520.3:c.1195G>A:p.(Glu399Lys) homozygous) showing very short and malformed limbs with redundant skin on arms and legs, short spade-like hands, narrow chest, absent mineralization of the cervical and thoracic bones, short “telephone handle” malformation of the femur, scanty mineralization of the skull bones, absent mineralization of tibiae and fibulae (died neonatally).

H) Terminated pregnancy of the sibling of 15DG1287 with the same phenotype.

Figure S3: Representative images of cases with lethal neurological disorders

A- MRI of 15DG0267 with holoprosencephaly (died neonatally).

B- CMA of 15DG0276 showing a large deletion on chromosome-13 that encompasses *ZIC2* and *ZIC5*.

C-D Clinical photograph & CT scan of 13DG0010, (*WDR81*:NM_001163809.1:c.845G>A: p.(Gly282Glu) homozygous) with holoprosencephaly showing dysmorphic facies, and excessive nuchal skin, (died neonatally). E-F) Brain MRI of 16DG0971 (*TRAK1*:NM_001042646.2:c.287-2A>G. homozygous) showing midbrain signal abnormality, mild dilation of the ventricles and diffuse brain atrophy.

G) Brain MRI of 15DG0227 (*POMT1*:NM_007171.3:c.280+1G>T homozygous) showing enlarged third and lateral ventricles with absence of septum pellucidum, absent corpus callosum, small posterior fossa with atrophy of vermis and cerebellar hemispheres and compression on the brainstem.

H- X-ray of 14DG0132 (*ISPD*:NM_001101426.3:c.790-1G>C:r.790_835del:p.(Val264Argfs*9) homozygous) showing microcephaly and myelomeningocele.

I- Brain MRI of 13DG2280 (*ASNS*: NM_001178076.1:c.962G>A:p.(Arg321His) homozygous) showing diffuse brain atrophy and simplified gyral pattern.

Figure S4: Representative images of cases with ciliopathy and metabolic phenotypes.

A-B) Clinical and radiographic images of 12DG2087 (*MKS1*:NM_017777.3:c.1126dupA:p.(Thr376Asnfs*3) homozygous) with MKS showing a very large encephalocele, massively enlarged abdomen (large polycystic kidneys) and lower-limb polydactyly. C) Clinical image of 16DG0474 (*CC2D2A*:NM_001080522.2:c.4531T>C :p.(Trp1511Arg) homozygous) with MKS showing encephalocele and bilaterally enlarged polycystic kidneys.

D -E) Clinical and radiographic images of 15DG2482 (*ACADVL*:NM_000018.3:c.65C>A: p.(Ser22*) homozygous) with VLCAD deficiency showing facial dysmorphism and cardiomegaly. F) Clinical image of 15DG1640 (*ABCA12*: NM_173076.2: deletion of exon-3) homozygous) showing harlequin ichthyosis.

Figure S5. Identification of *TNNT3*-related lethal phenotype.

A) Pedigree of 21DG0001 (*TNNT3*:NM_006757.4:c.723-2A>G homozygous) with NIHF and neonatal death.

B-E) Antenatal ultrasound images at 22 weeks showing a thick nuchal fold (B), scalp edema and micrognathia (C), clenched hands bilaterally (D) and fixed extended lower limbs (E).

Figure S6. Identification of *LZTR1*-related lethal phenotype (autosomal recessive).

A) Pedigree of 16DG1276 (*LZTR1*:NM_006767.4:c.2317G>A:p.(Val773Met) homozygous) with stillbirths and neonatal deaths.

B) Antenatal ultrasound at 12 weeks showing massive skin edema (terminated).

Figure S7. Identification of *MYH11*-related lethal phenotype (autosomal recessive).

A) Pedigree of 17DG1094 (*MYH11*:NM_022844.3:c.1033+1G>A homozygous) with recurrent stillbirths.

B-E) Antenatal ultrasound showing dilated kidneys, ascites, dilated bladder, and talipes respectively.

Figure S8. Identification of *FGFR3*-related lethal phenotype (homozygous).

A) Pedigree of F8317 (*FGFR3*:NM_000142.3:c.1138G>A: p.(Gly380Arg) homozygous)

B) CT scan of the chest showing bilateral diffuse opacities more in the lower lobes, dilated esophagus. C) X-ray showing narrow chest and acetabular dysplasia.

Figure S9. Identification of a *BCS1L*-related lethal phenotype by RT-PCR.

A) Pedigree of 11DG1647 with recurrent lactic acidosis and neonatal death.

B) Sequence chromatogram of *BCS1L*:NM001079866:c.441C>T variant.

C and D) RT-PCR of *BCS1L* showing aberrant splicing and skipping of 23bp at end of exon-3.
